# mTORC1 Inhibition Protects Human Regulatory T Cells From Granzyme-B-Induced Apoptosis

**DOI:** 10.3389/fimmu.2022.899975

**Published:** 2022-06-10

**Authors:** Siawosh K. Eskandari, Hazim Allos, Basmah S. Al Dulaijan, Gandolina Melhem, Ina Sulkaj, Juliano B. Alhaddad, Anis J. Saad, Christa Deban, Philip Chu, John Y. Choi, Branislav Kollar, Bohdan Pomahac, Leonardo V. Riella, Stefan P. Berger, Jan S. F. Sanders, Judy Lieberman, Li Li, Jamil R. Azzi

**Affiliations:** ^1^ Transplantation Research Center, Division of Nephrology, Brigham and Women’s Hospital, Harvard Medical School, Boston, MA, United States; ^2^ Division of Nephrology, University Medical Center Groningen, University of Groningen, Groningen, Netherlands; ^3^ Graduate Program in Immunology, Johns Hopkins School of Medicine, Baltimore, MD, United States; ^4^ Division of Plastic Surgery, Brigham and Women’s Hospital, Harvard Medical School, Boston, MA, United States; ^5^ Department of Plastic and Hand Surgery, University of Freiburg Medical Center, Medical Faculty of the University of Freiburg, Freiburg, Germany; ^6^ Division of Plastic and Reconstructive Surgery, Smilow Cancer Hospital, Yale School of Medicine, New Haven, CT, United States; ^7^ Center of Transplantation Sciences, Division of Nephrology, Massachusetts General Hospital, Harvard Medical School, Charlestown, MA, United States; ^8^ Program in Cellular and Molecular Medicine, Boston Children’s Hospital, Harvard Medical School, Boston, MA, United States; ^9^ Department of Medical Oncology, Dana-Farber Cancer Institute, Harvard Medical School, Boston, MA, United States; ^10^ Division of Nephrology, Brigham and Women’s Hospital, Harvard Medical School, Boston, MA, United States

**Keywords:** granzyme B, mTORC1, rapamycin, grapoptosis, human tregs, treg homeostasis

## Abstract

Regulatory T cells (T_regs_) have shown great promise as a means of cellular therapy in a multitude of allo- and auto-immune diseases—due in part to their immunosuppressive potency. Nevertheless, the clinical efficacy of human T_regs_ in patients has been limited by their poor *in vivo* homeostasis. To avert apoptosis, T_regs_ require stable antigenic (CD3ζ/T-cell-receptor-mediated), co-stimulatory (CD28-driven), and cytokine (IL-2-dependent) signaling. Notably, this sequence of signals supports an activated T_reg_ phenotype that includes a high expression of granzymes, particularly granzyme B (GrB). Previously, we have shown that aside from the functional effects of GrB in lysing target cells to modulate allo-immunity, GrB can leak out of the intracellular lysosomal granules of host T_regs_, initiating pro-apoptotic pathways. Here, we assessed the role of inhibiting mechanistic target of rapamycin complex 1 (mTORC1), a recently favored drug target in the transplant field, in regulating human T_reg_ apoptosis *via* GrB. Using *ex vivo* models of human T_reg_ culture and a humanized mouse model of human skin allotransplantation, we found that by inhibiting mTORC1 using rapamycin, intracytoplasmic expression and functionality of GrB diminished in host T_regs_; lowering human T_reg_ apoptosis by in part decreasing the phosphorylation of S6K and c-Jun. These findings support the already clinically validated effects of mTORC1 inhibition in patients, most notably their stabilization of T_reg_ bioactivity and *in vivo* homeostasis.

**Graphical Abstract:**
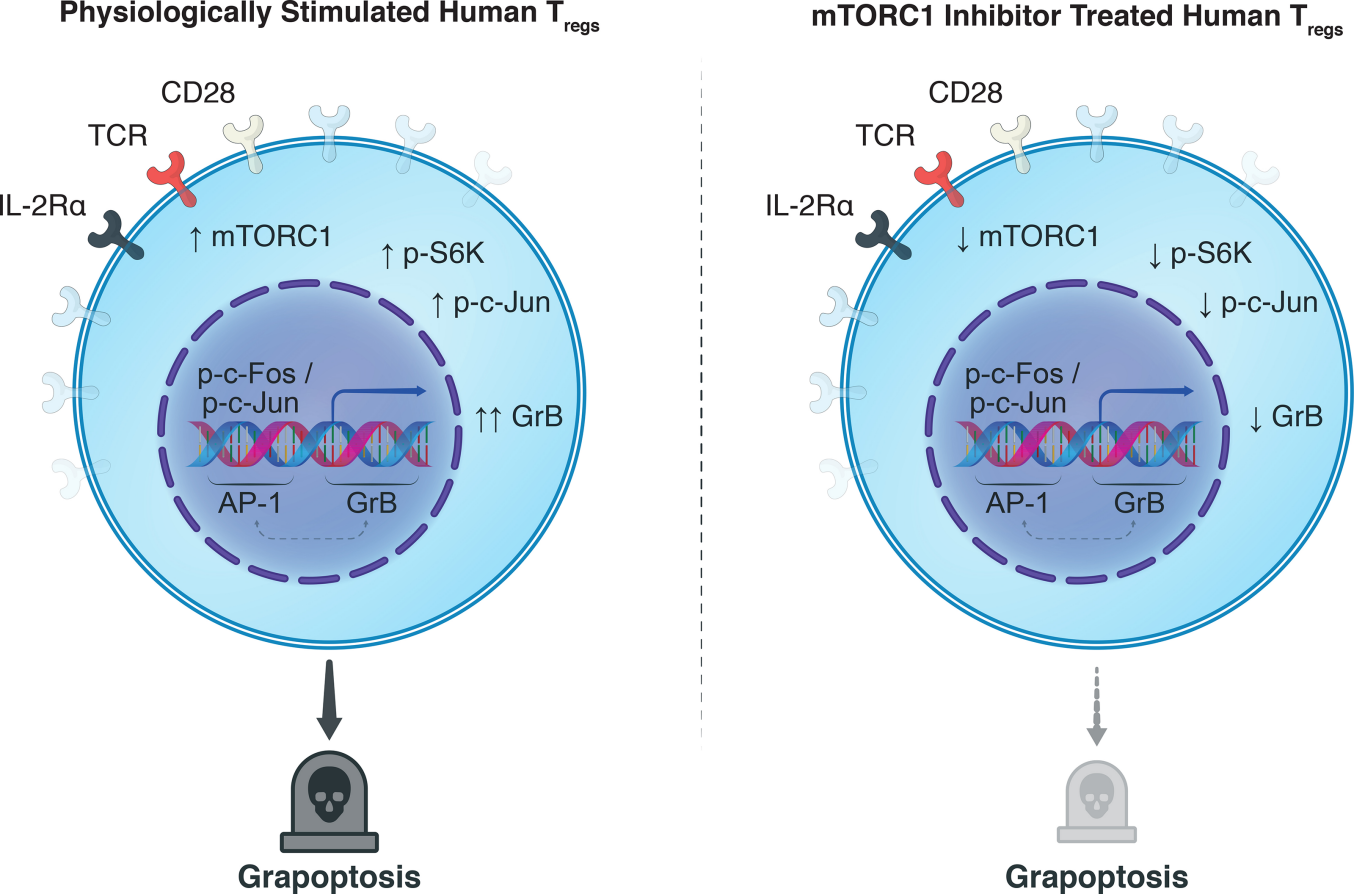


## Introduction

The first description of cells residing in the lymphatics with an immunoregulatory phenotype dates back to studies by Gershon et al. in 1972 (1). The formal phenotypic characterization of what we now know as CD4^+^ regulatory T cells (T_regs_), however, was not established until 1995 and 2003, when interleukin 2 receptor alpha (IL-2Rα; CD25) and FoxP3 were respectively identified as essential T_reg_ markers in mice (2–4). In humans, additional studies later found that IL-7Rα (CD127) expression on CD4^+^ T cells inversely correlated with the human T_reg_ phenotype, such that CD4^+^ CD25^high^CD127^low^ T cells marked the purest human T_reg_ population (5, 6). IL-7 is notably a memory cytokine that promotes the survival of human CD4^+^ T cells (7), where it is hypothesized that we humans acquire specialized memory CD4^+^ CD25^hi^CD127^hi^ non-T_regs_ over the course of our lives in response to a variety of serendipitous pathogen exposures. Since the discovery of human CD4^+^ CD25^hi^CD127^lo^ FoxP3^+^ T_regs_, a myriad of clinical applications have been explored for auto-immune diseases and organ and tissue transplantation with varying degrees of success (8).

In general, therapeutic strategies involving T_regs_ begin with the isolation of T_regs_ from the peripheral blood of a patient, followed by an ex vivo expansion protocol, and ultimately adoptive re-infusion back into the patient (9, 10). Nonetheless, despite evolving techniques in isolating, handling, and expanding T_regs_, T_reg_ immunotherapy continues to be thwarted by invariably short T_reg_ lifespan in patients and the unstable expression of the transcriptional immunoregulator FoxP3 (11). Additionally, considering the plasticity of the T_reg_ phenotype, a worrisome complication of T_reg_ therapy is the conversion of T_regs_ into pathologic T-cells secreting pro-inflammatory cytokines such as IL-17, which notoriously mediate auto-immune processes (12).

One avenue of optimizing T_reg_ therapy involves studying the physiological working mechanisms of T_regs_ to allow for engineered T_regs_ that can withstand unfavorable *in vivo* milieus. Notably, T_regs_ employ a host of cell-contact dependent and independent methods to induce immunosuppression, where each immunoregulatory method is reliant on circumstantial factors such as the target cells, the context of immune response, and the anatomical site of suppression. Among these mechanisms of T_reg_ action, granzyme B (GrB-)mediated cytolysis (also, granolysis) of target cells is one of the vital pathways with which T_regs_ block effector T-cell proliferation (13, 14). While GrB is essential in targeting effector cells, however, our group has previously shown that this serine protease can also play a role in destabilizing host T_regs_, thus, predisposing them to granzyme-B-induced apoptosis (grapoptosis) (15, 16). Particularly, we found that GrB, which is physiologically stored in intracytoplasmic lysosomal granules prior to being exocytosed for targeted lysis, can leak from these granules upon T-cell-receptor-dependent activation and precipitate both caspase dependent and independent cell death (16). The exact mechanisms underlying granzyme leakage from the lysosomal granules remain to be fully understood.

Separate studies looking at novel drug-based immunotherapies have recently led to the understanding that there are T_reg_-favorable and -unfavorable immunosuppressants depending on the drugs’ impact on the T_reg_ compartment (17, 18). With regards to the T_reg_-favorable drugs, a growing body of evidence attributes T_reg_-sparing qualities to inhibitors of mechanistic target of rapamycin complex 1 (mTORC1) such as rapamycin (also known as sirolimus)—although the mechanisms behind this effect are incompletely understood (19).

Here, we studied the interplay between grapoptosis and mTORC1 signaling in human T_regs_. Notably, using the mTORC1 inhibitor rapamycin, we observed an intrinsic link between the mTORC1 pathway and GrB activity in which mTORC1 inhibition reduced intracellular GrB reserves and spared T_reg_ viability.

## Methods

### Materials

Rapamycin solution (#S-015) and cyclosporin A solution (#C-093) were both purchased from Millipore Sigma (Burlington, MA, USA). Recombinant human IL-2 (#212-12) was purchased from PeproTech (Rocky Hill, NJ, USA). Soluble human anti-CD3 (OKT3, #16-0037-85) and soluble human anti-CD28 (28.2, # 16-0289-85) were purchased from Invitrogen (Carlsbad, CA, United States).

### Human Samples

Leukapheresed blood from healthy individuals was obtained from the Brigham and Women’s Hospital Blood Bank. Blood samples were processed for peripheral blood mononuclear cell (PBMCs) isolation within 24 hours by SepMate tubes (#85450, STEMCELL Technologies).

Healthy skin was obtained from consenting patients undergoing cosmetic procedures at the Brigham and Women’s Hospital Plastic Surgery department. The study protocol was approved by an Institutional Review Board at the Brigham and Women’s Hospital and was performed in accordance with the principles of the Declaration of Helsinki.

### Cell Culture

Cells were cultured in complete Dulbecco’s Modified Eagle’s medium (#10-013-CV, Corning) supplemented with 9% pooled GemCell U.S. Origin Human Serum AB (#100-512, GeminiBio), 2 mM L-glutamine (#25030081, Gibco), and 1% penicillin-streptomycin (#15140122, Gibco).

Functional grade purified anti-human CD3 (OKT3) and functional grade purified anti-human CD28 (CD28.2; eBioscience) were added in suspension at a concentration of 4 μg/mL together with 200 ng/mL recombinant human IL-2 (PeproTech).

### Statistics

Differences between two normally distributed groups were analyzed with independent samples two-tailed Student t-tests, and non-parametric Mann-Whitney U-tests when the assumption of homoscedasticity could not be met. Statistical analyses of multiple groups were performed with one-way analyses of variance followed by Holm-Šídák multiple comparison tests, or mixed-effects model analyses with the Geisser-Greenhouse correction followed by Holm-Šídák multiple comparison tests for experimental groups with matched data points across multiple time points or concentrations. *P* < 0.05 was considered significant for all analyses. Data analysis and graphing were performed with Prism 9.3.1 (GraphPad Software). Graphs show boxplots with median, interquartile range, minimum, maximum, and all individual data points of the denoted experimental groups.

Additional experimental details are described in the **Supplementary Methods**.

## Results

### mTORC1 Inhibition Reduces GrB Expression and Improves T_reg_ Viability

While an expanding fund of knowledge has linked granzyme (GrB) production, mTORC1 signaling, and CD8^+^ T cell activation (20–22)—cytotoxic CD8^+^ T cells being known for producing vast quantities of GrB (23)—it was unknown if the same intrinsic link existed in T_regs_. First, to recapitulate earlier findings that human T_regs_ upregulate GrB and markers of apoptosis (16), we isolated human CD4^+^ CD25^hi^CD127^lo^ T_regs_ from the peripheral blood mononuclear cells (PBMCs) of healthy donors (**Figures S1A–C**). Next, we expanded them *ex vivo* for three days in the presence of α-CD3, α-CD28, and IL-2—thus, providing antigenic, co-stimulatory, and cytokine signals. Importantly, throughout our experiments we opted for a minimum viable cell culture medium (see *Methods*) as opposed to an optimized T_reg_ medium (24) to obviate potential compensatory and confounding glycolytic and metabolic effects exacted by exogenous cell culture reagents such as non-essential amino acids. This was most relevant for the experiments including mTORC1 inhibition, due to mTORC1’s role in both the immune system and cellular metabolism (23). Using flow cytometry to identify the activated CD4^+^ CD25^hi^CD127^lo^ FoxP3^+^ T_reg_ subset (**Figures S2A, B**), we observed 36.9% GrB^+^ and 39.0% Annexin V^+^ expressing T_regs_ on average (**Figures S2C, D**). Importantly, staining the cell surface with Annexin V is a sensitive method for measuring the rate of apoptosis in many cell types including T cells (25). Further bisecting the T_reg_ population into GrB^-^ and GrB^+^ subsets, we confirmed that the GrB^+^ T_regs_ were characterized by an increased rate of Annexin V^+^ apoptosis compared to their GrB^-^ counterparts (16.6% vs. 82.9%, GrB^-^ vs. GrB^+^ T_regs_, *P* < 0.0001; **Figures S2E, F**).

**Figure 1:**
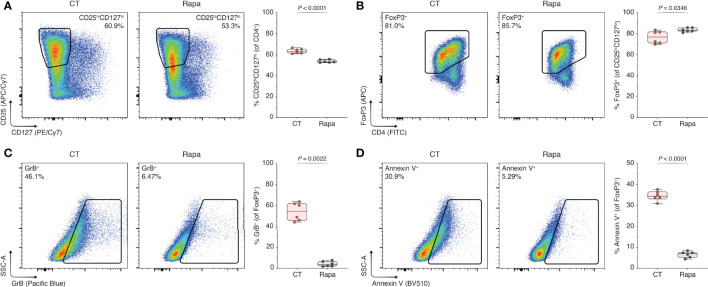
mTORC1 inhibition prevents GrB upregulation and rescues T_regs_ from apoptosis. Human T_regs_ were isolated and expanded for three days with α-CD3, α-CD28, and IL-2, without additional inclusions (CT), or with CT stimulants plus 100 nM rapamycin (Rapa). **(A–D)** Flow cytometric analyses of CD25^hi^CD127^lo^ subset among CD4^+^ T cells **(A)**, FoxP3^+^ subset among CD25^hi^CD127^lo^ cells **(B)**, and GrB^+^ **(C)** and Annexin V^+^ subsets **(D)** among FoxP3^+^ T_regs_, including the respective box plots (n = 3 technical replicates/condition; 2 representative experiments of 5). Data represent boxplots with median, interquartile range, minimum, maximum, and all individual data points of the denoted experimental groups. *P* values were calculated with independent samples two-tailed Student’s *t*-tests, and non-parametric Mann-Whitney *U*-tests were performed when the assumption of homoscedasticity could not be met. CT, control; GrB, granzyme B; Rapa, rapamycin.

To assess the effect of mTORC1 inhibition on T_regs_, GrB production, and the rate of T_reg_ apoptosis, we repeated the experiments in the presence of the specific mTORC1 inhibitor rapamycin, where rapamycin is known as a potent immunosuppressant with T_reg_-sparing effects (26–28). Using flow cytometry **Figure S3A**), we observed a reduction in the CD25^hi^CD127^lo^ population of CD4^+^ T cells in the context of rapamycin treatment (63.4% vs. 53.7%, control (CT) vs. rapamycin (Rapa), *P* < 0.0001; **Figure 1A**). Conversely, Rapa treatment yielded an increase in the FoxP3^+^ subset (76.5% vs. 83.5%, CT vs. Rapa, *P* = 0.0346; **Figure 1B**). Within the FoxP3^+^ T_reg_ population, we found that the increase in GrB expression in CT T_regs_ was abated with Rapa treatment (54.0% vs. 4.14%, CT vs. Rapa, *P* = 0.0022; **Figure 1C**) as well as the rate of apoptosis (34.5% vs. 6.55%, CT vs. Rapa, *P* < 0.0001; **Figure 1D**). Besides reduced percentages of GrB and Annexin V, the mean fluorescence intensities (MFIs) of GrB and Annexin V were also dimmed in the Rapa-treated T_regs_ compared to the CT T_regs_ **Figures S3B, C**). Synthesizing the decreased CD25^hi^CD127^lo^ expression, increased Foxp3 percentage, and reduced Annexin V positivity, we found that Rapa treatment yielded 10% more viable CD4^+^ CD25^hi^CD127^lo^ FoxP3^+^ T_regs_ in our ex vivo model compared to the regularly treated CT T_regs_ (31.8% vs. 41.9%, CT vs. Rapa, *P* < 0.0001; **Figure S3D**)—these combined effects potentially explaining rapamycin’s “T_reg_-sparing” moniker.

To understand the potential dose-dependency of rapamycin’s effect on the T_reg_ compartment, we de-escalated our standard ex vivo rapamycin dose of 100 nM in ^10^Log steps, yielding a treatment of range of 0.1–10 nM of Rapa. Additionally, to ascertain whether the effects of rapamycin on GrB production and Annexin V apoptosis were due to the decreased activation of T_regs_ per se rather than a unique mTORC1-specific mechanism of action, we treated a group of human T_regs_ with cyclosporin A (CsA). Importantly, CsA belongs to a drug class known as calcineurin inhibitors (CNIs), which are archetypal immunosuppressants that continue to be used for solid organ transplantation to this day (28, 29). Using flow cytometry as described earlier **Figure S3A**), we found that the reduction in CD25^hi^CD127^lo^ and increase in FoxP3 T cells indeed depended on the Rapa dose, however, not attaining statistical significance in increasing the FoxP3^+^ percentage in the range of 0.1–10 nM Rapa as with the 100 nM dosage **Figures S4A, B**). Treatment of T_regs_ with CsA decreased CD25^hi^CD127^lo^ and increased FoxP3 as with Rapa treatment, but also without attaining statistical significance **Figures S4A, B**). Looking at the GrB expression in the FoxP3^+^ T_regs_, intriguingly, we found that Rapa de-escalation significantly increased the GrB percentages and MFIs, returning them to the level of the CT T_regs_ at the lowest Rapa dose **Figure S4D**). On the contrary, CsA treatment did not diminish GrB expression, in fact, even increasing it (43.9% vs. 55.8%, CT vs. CsA, *P* = 0.0126; **Figure S4D**). Regarding the rate of apoptosis, Rapa de-escalation again resulted in a return to the CT T_reg_ levels, while CsA treatment marginally decreased the Annexin V expression compared to CT Tregs (28.6% vs. 20.3%, CT vs. CsA, *P* = 0.0041; **Figure S4E**).

### mTORC1 Inhibition Rescues T_regs_ From Intracytoplasmically Active GrB

**Figure 2:**
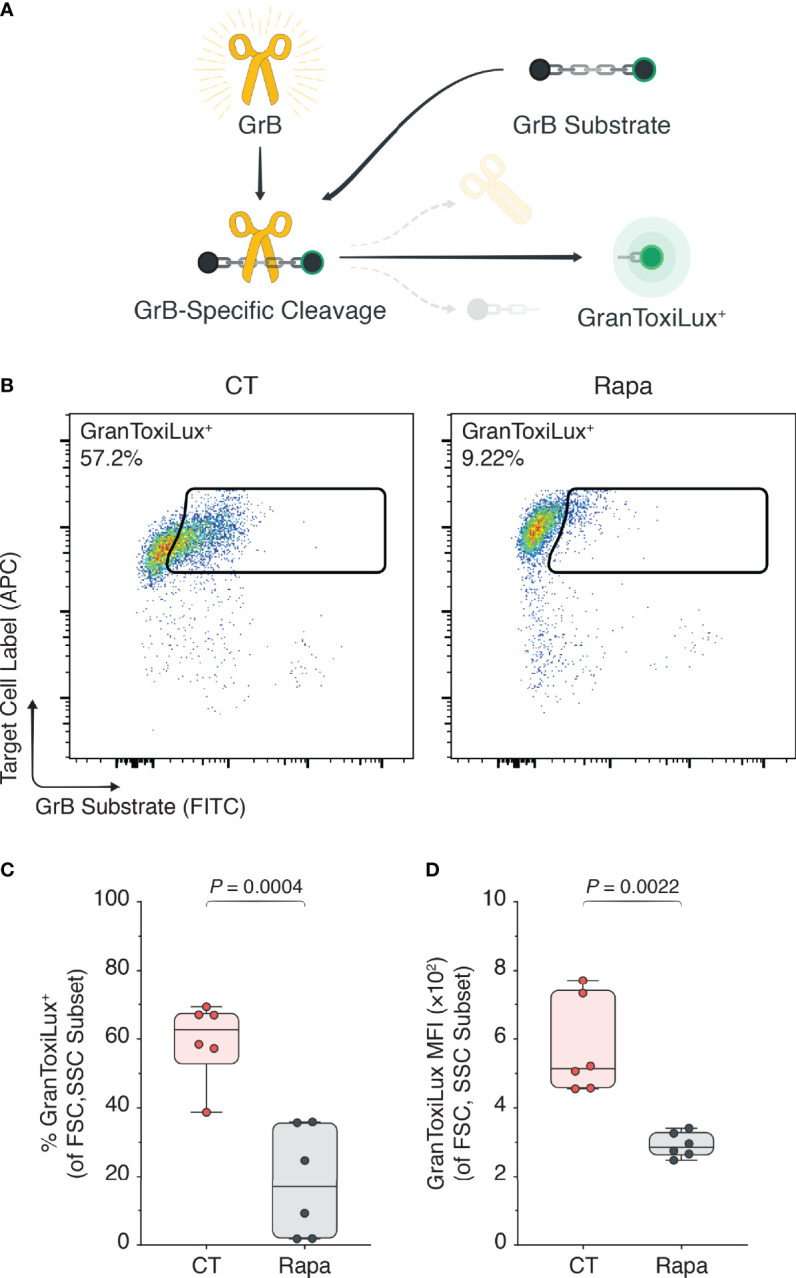
mTORC1 inhibition attenuates intracytoplasmically active GrB. Human T_regs_ were isolated and expanded for three days with α-CD3, α-CD28, and IL-2 (CT), or with CT stimulants plus 100 nM rapamycin (Rapa). **(A)** The GranToxiLux assay was used to determine functionally active GrB in the cytoplasmic T_reg_ compartment, where active GrB cleaves the GrB-sensitive GranToxiLux substrate to yield a fluorescent GranToxiLux reagent detectable in the FITC channel. **(B)** Flow cytometric plots of active GrB in the T_reg_ cytoplasm. **(C, D)** Box plots of GranToxiLux^+^ percentages **(C)** and mean fluorescence intensities **(D)** among the T_reg_ population (n = 3 technical replicates/condition; 2 representative experiments of 4). Data represent boxplots with median, interquartile range, minimum, maximum, and all individual data points of the denoted experimental groups. *P* values were calculated with independent samples two-tailed Student’s *t*-tests, and non-parametric Mann-Whitney *U*-tests were performed when the assumption of homoscedasticity could not be met. CT, control; GrB, granzyme B; MFI, mean fluorescence intensity; Rapa, rapamycin; T_regs_, regulatory T cells.

Next, we sought to determine if mTORC1 inhibition through rapamycin decreased GrB expression in activated T_regs_ per se or if it also affected the functionally active GrB escaping from the lysosomes into the cytoplasm. To measure intracytoplasmic GrB activity we used a specialized GranToxiLux assay (16, 30), which permits measurement of active GrB through surrogate green fluorescence emission. Importantly, green fluorescence is only emitted when the GrB-sensitive substrate in the GranToxiLux assay is cleaved (**Figure 2A**). To validate the GranToxiLux assay, we first isolated human CD4^+^ CD25^hi^CD127^lo^ T_regs_ from the PBMCs of healthy donors and expanded them ex vivo for three days in the presence of α-CD3, α-CD28, and IL-2. Using flow cytometry **Figure S5A**), we identified the blastic (FSC^hi^SSC^hi^) lymphocyte subset, observing a significant increase in both the percentages of intracytoplasmically active GrB in the activated T_reg_ condition compared to the freshly isolated, naïve T_regs_ (4.85% vs. 45.2%, naïve vs. activated T_regs_, *P* < 0.0001; **Figures S5B, C**) as well as the MFIs (180 vs. 313, naïve vs. activated T_regs_, *P* < 0.0001; **Figure S5D**).

To appreciate the impact of rapamycin on the GranToxiLux substrate conversion, we repeated the experiments in the presence of rapamycin with T_regs_ isolated from new PBMC donors. We observed a significant attenuation of the percentages of active GrB in the cytoplasm of Rapa-treated T_regs_ compared with CT T_regs_ (59.58% vs. 18.21%, naïve vs. activated T_regs_, *P* = 0.0004; **Figures 2B, C**) as well as the GranToxiLux substrate MFIs (574 vs. 292, naïve vs. activated T_regs_, *P* = 0.0022; **Figure 2D**).

### mTORC1 Inhibition Impedes Signalling Through p-S6K and p-c-Jun

**Figure 3:**
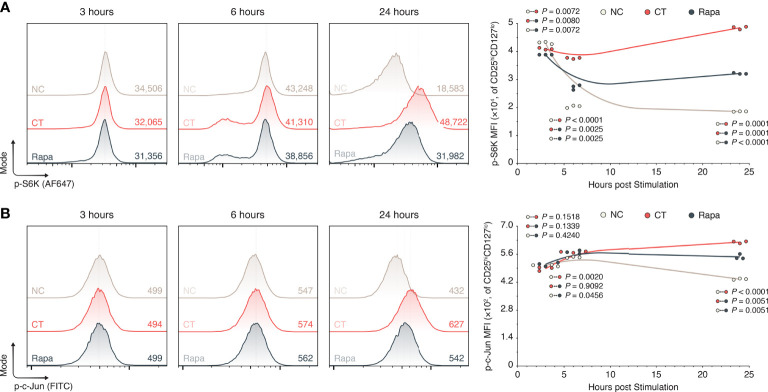
mTORC1 inhibition attenuates S6K and c-Jun phosphorylation. Human T_regs_ were isolated and expanded in a 24-hour cell culture window without inclusions (NC), with α-CD3, α-CD28, and IL-2 (CT), or with CT stimulants and 100 nM rapamycin (Rapa). **(A)** Flow histograms of p-S6K expression among CD25^hi^CD127^lo^ T_regs_, including inset mean fluorescence intensities (MFIs) and the 24-hour period XY plot (n = 3 technical replicates/condition/timepoint; 1 representative experiment of 3). **(B)** Flow histograms of p-c-Jun expression among CD25^hi^CD127^lo^ T_regs_, including inset mean fluorescence intensities (MFIs) and the 24-hour period XY plot (n = 3 technical replicates/condition/timepoint; 1 representative experiment of 3). All individual datapoints are displayed. *P* values were calculated using mixed-effects model analyses with the Geisser-Greenhouse correction followed by Holm-Šídák multiple comparison tests. AF647, Alexa Fluor 647; CT, control; MFI, mean fluorescence intensity; NC, negative control; Rapa, rapamycin.

To explore the potential mechanistic link between mTORC1 and GrB, we studied the expression of phosphorylated S6 kinase 1 (p-S6K) and c-Jun (p-c-Jun) in human CD4^+^ CD25^hi^CD127^lo^ T_regs_ isolated from the PBMCs of healthy donors. These particular targets were chosen on the basis that mTORC1 physiologically endorses its pleiotropic effects through phosphorylation of S6K—among other kinases— (31, 32) and GrB is known to be upregulated by a variety of transcription factors including activating protein (AP-)1, a heterodimer consisting of proteins including c-Jun (33). We cultured the isolated T_regs_ ex vivo in a 24-hour window without stimulants (NC), or with α-CD3, α-CD28, and IL-2 in the presence or absence of rapamycin (Rapa and CT respectively). Using flow cytometry **Figure S6**), we found that providing 24 hours of mitogenic stimuli to T_regs_ increased their expression of p-S6K relative to the non-stimulated T_regs_ (1.86×10^4^ vs. 4.85×10^4^, NC vs. CT T_regs_, *P* = 0.0001; **Figure 3A**) as well as their expression of p-c-Jun (435 vs. 623, NC vs. CT T_regs_, *P* < 0.0001; **Figure 3B**). Addition of rapamycin to the human T_reg_ culture, however, impeded the upregulation of p-S6K relative to the CT T_regs_ as early as 6 hours post treatment (3.76×10^4^ vs. 2.72×10^4^, CT vs. Rapa T_regs_, *P* = 0.0025; **Figure 3A**) and within 24 hours of treatment for the upregulation of p-c-Jun (623 vs. 550, CT vs. Rapa T_regs_, *P* = 0.0051; **Figure 3B**).

### mTORC1 Inhibition Improves Human T_reg_ Homeostasis in an Allo-Immune Context

Although ex vivo experiments provide great insights into the working mechanisms of T_reg_-affecting drugs, we also sought to investigate if mTORC1 inhibition could affect GrB and Annexin V expression of human T_regs_ in an *in vivo* humanized mouse model (**Figure S7A**). In this model, we transplanted healthy human skin obtained from consenting patients undergoing cosmetic surgery onto the trunks of NOD-*scid* IL-2 receptor-γ^null^ (NSG) mice. Seven days post-transplant, 5.0×10^6^ human PBMCs and 1.0×10^6^ human T_regs_ were adoptively transferred into the NSG mice to respectively initiate and suppress the allo-immune responses (**Figure S7B**). To assess the effects of mTORC1 inhibition on the T_regs_ we compared daily intraperitoneal injections of the phosphate-buffered saline vehicle (PBS) to 1 mg/kg rapamycin injections. Twenty-one days post transplantation, we euthanized the mice and studied the splenocytes by flow cytometry (**Figure S7B**). Notably, this model has several limitations. First, the read-out sites are restricted to the splenic compartment as T_regs_ from the skin grafts cannot be extracted from the dermal-epidermal interface without disrupting the T_reg_ surface phenotype—due to the required enzymatic digestion—and there are no secondary lymphoid tissues (SLT) apart from the spleen due to SLT atrophy in the NSG mice. Secondly, there are methodological restrictions to attaining large enough human T_reg_ quantities for survival studies as well as being able to include a multitude of conditions. Nevertheless, this model does allow us to study the effect of rapamycin on T_regs_ in four dimensions, namely in three-dimensional space and over time, permitting the assessment of GrB expression and Annexin V positivity on *in vivo* derived human T_regs_.

Using flow cytometry to identify the human T_regs_ residing within in the spleen (**Figures S7C, D**), we found that rapamycin treatment marginally increased the percentage of CD4^+^ CD25^+^FoxP3^+^ T_regs_ relative to PBS-treated T_regs_, but not significantly so (34.6% vs. 47.3%, PBS vs. Rapa, *P* = 0.0959; **Figure S7D**). Additionally, looking at the CD4^+^ CD25^++^FoxP3^+^ T_reg_ subset we found that rapamycin treatment did not affect this subset relative to the PBS treatment group (2.1% vs. 2.3%, PBS vs. Rapa, *P* = 0.8678; **Figure S7D**). At the same time, however, the GrB expression among the splenic T_regs_ of the Rapa-treated mice was significantly lowered compared to the splenic PBS T_regs_ (53.4% vs. 34.1%, PBS vs. Rapa, *P* = 0.0043; **Figures S7E, F**). Additionally, the Annexin V expression in the Rapa condition was significantly attenuated compared to the PBS condition (56.0% vs. 33.8%, PBS vs. Rapa, *P* = 0.0042; **Figures S7G, H**). Thus, although the percentages of CD4^+^ CD25^+^FoxP3^+^ T_regs_ were not significantly different between the treatment groups, looking at the viable CD4^+^ CD25^+^FoxP3^+^ T_regs_, we found significantly more live T_regs_ in the Rapa condition compared to the PBS group (16.7% vs. 32.5%, PBS vs. Rapa, *P* = 0.0123; **Figure S7I**). Finally, to assess if the observed effects in the splenic compartment of the Rapa-treated group were due to mTORC1 inhibition, we stained the splenocytes for phosphorylated 4E-BP. Notably, the phosphorylation of 4E-BP is physiologically repressed by mTORC1 (32), thus, expecting p-4E-BP to be increased in mTORC1-inhibited cells. Indeed, we found that the splenic T_regs_ of the Rapa-treated mice expressed higher percentages of p-4E-BP compared with the T_regs_ of PBS-treated mice (28.8% vs. 50.1%, PBS vs. Rapa, *P* = 0.0269; **Figure S7J**), indicating a veritable effect of the Rapa treatment on these T_regs_.

## Discussion

A growing body of evidence supports the influence of granzyme (GrB) secretion by activated T_regs_ on the suppression of effector lymphocytes and regulation of overall allograft tolerance (13–15, 34). While GrB is vital for the effector functionality of T_regs_, however, we recently showed that activated T_regs_ also have the propensity to leak GrB in the intracytoplasmic compartment of host T_regs_, leading to caspase-3 dependent and independent cell death (16). This finding also proved clinically relevant, as we found that T_regs_ in the peripheral blood of renal allografts recipients with T-cell-mediated rejection expressed higher levels of GrB—thus, making them more prone to granzyme-B-induced apoptosis (grapoptosis) (16). Since the poor *in vivo* homeostasis of T_regs_ handicaps the clinical application of these cells as an immunosuppressive cytotherapy, insights on the factors that moderate T_reg_ survival are much needed to improve the efficacy of adoptively transferred T_regs_ in patients (35). Here, we explored the potential role of GrB in regulating T_reg_ homeostasis in association with mTORC1 signaling, considering recent lines of inquiry have attributed T_reg_-sparing qualities to mTORC1 inhibitors (36, 37). mTORC1 inhibitors notably suppress pro-inflammatory T cells while sparing or in certain cases even inducing proliferation of T_regs_, making them an attractive target to study in the context of grapoptosis.

While several studies in the literature have previously hinted at mTORC1 inhibitors reducing GrB expression in activated T_regs_ (15, 16, 34, 38), the relationship between decreased GrB expression and increased viability was not yet explored. In our experiments, we found that mTORC1 inhibition could indeed spare T_reg_ homeostasis by decreasing both intracellular GrB expression and activity, and protect T_regs_ from pro-apoptotic pathways marked by the expression of Annexin-V-specific phospholipids on the T_reg_ surface. Accordingly, we speculate that the treatment of patients with mTORC1 inhibitors could be lowering the apoptotic rate of T_regs_ by, in part, decreasing intracytoplasmic GrB activity.

Intriguingly, while the treatment of human T_regs_ with rapamycin in our ex vivo models lead to reduced GrB levels and increased viability, we did not observe the same effect upon treating human T_regs_ with the calcineurin inhibitor (CNI) cyclosporin A. CNIs are notably a mainstay of the immunosuppressive regime used after solid organ transplantation (28), realizing immunosuppression in a T cell agnostic fashion by eliminating both T_regs_ and T_cons_, and possibly even inhibiting T_regs_ to a greater degree (15, 34, 38). mTORC1 inhibitors, in contrast, selectively downregulate the serine/threonine kinase mTOR—which physiologically promotes the survival, proliferation, and accentuation of T cell function downstream of the T-cell-receptor (TCR), CD28, and IL-2 signaling pathways (27, 39, 40). Mounting evidence now suggests that mTORC1 inhibitors preferentially suppress T_con_ proliferation over that of T_regs_, although rapamycin is not agnostic to the T_reg_ population and can similarly inhibit T_regs_ at higher concentrations (41). Notably, however, T_regs_ can alternatively rely on different kinases for cell growth and activation, thus maintaining their activity and proliferative capacity despite mTORC1 inhibition (26–28). We hypothesize that T_regs_ may possess these redundancies to mTORC1 signaling to be able to survive in a wide range of milieus, including competitive niches such as inflammatory sites and the tumor microenvironment. Notably, T_regs_ can metabolically adapt to these harsh environments by switching to aerobic glycolysis, upregulating FoxP3 in response to hypoxia, and outcompeting other T cells subsets under acidic, low glucose, and high lactate conditions (42–44). In fact, it seems T_regs_ have not only evolved to survive reducing environments but are now even known to employ extracellular redox metabolites to suppress effectors T cells (45). Considering mTORC1 sits at the interface of immune responses and metabolic activities—a burgeoning domain known as immunometabolism—mTORC1 inhibition is perhaps only likely to affect T_reg_ homeostasis. Indeed, in clinical trials with kidney and transplant recipients, mTORC1 inhibitors benefitted T_reg_ counts in the peripheral circulation when compared to CNIs (46, 47), while in another trial, liver transplant recipients who were converted from tacrolimus to sirolimus—a CNI to an mTORC1 inhibitor—demonstrated elevated numbers of T_regs_ in their blood and liver grafts (48).

Unsurprisingly, mTORC1 inhibitors such as sirolimus and everolimus have earned the reputation of being T_reg_-friendly compared to alternative immunosuppressive strategies (49). The effect of mTORC1 inhibition on T_regs_, however, is not entirely unambiguous. Experiments by Procaccini and colleagues have previously indicated that mTORC1 is locked in a complex, oscillatory dance with T_regs_—mTORC1 being a context-dependent regulatory checkpoint for CD4^+^ CD25^hi^CD127^lo^ T_reg_ activity, proliferation and fate (36). Human T_regs_ can even express elevated levels of phosphorylated translational regulators of mTORC1 including p70S6K and S6 compared to the effector CD4^+^ CD25^lo^ T cell population (18, 36). This finding was further elaborated on with mice deficient in Raptor, an mTORC1 adaptor protein, which displayed disrupted T_reg_ responses—indicating a contextually protective role for mTORC1 in certain autoimmune diseases (18). Additionally, work by Sun and colleagues showed that mTORC1 is highly expressed in the effector cluster of murine T_regs_ compared to the “resting reservoir” of central T_regs_, positing that mTORC1 activity is required for the conversion of central T_regs_ to effector T_regs_ and can be inhibited with rapamycin (50). Considering the potential disparate roles of mTORC1 in the murine T_reg_ compartment, further research towards the relevance of the murine findings in human T_regs_ and the role of mTORC1 in the different T_reg_ subsets is warranted.

Regarding the specific interplay between mTORC1 and GrB it is worthy to note that these axes have previously been linked in cytotoxic CD8 T cells (CTLs) (20–22). Notably, GrB is highly expressed by CTLs, being the tenth most produced protein by CTLs overall (23), while the overexpression (22) and downregulation (22, 23) of mTORC1 in CD8 T cells respectively resulted in the upregulation and attenuation of GrB. Taking an excursion to the disparate domain of fruit flies, a recent study by Jouandin and colleagues in *Science* interestingly tied the lysosomal compartment and TORC1 responses together (51). While this study did not explore the link between GrB stores in the lysosomes and TORC1, it did intrinsically link the autolysosome to the latter, reiterating the possible connection between GrB and mTORC1.

**Figure 4:**
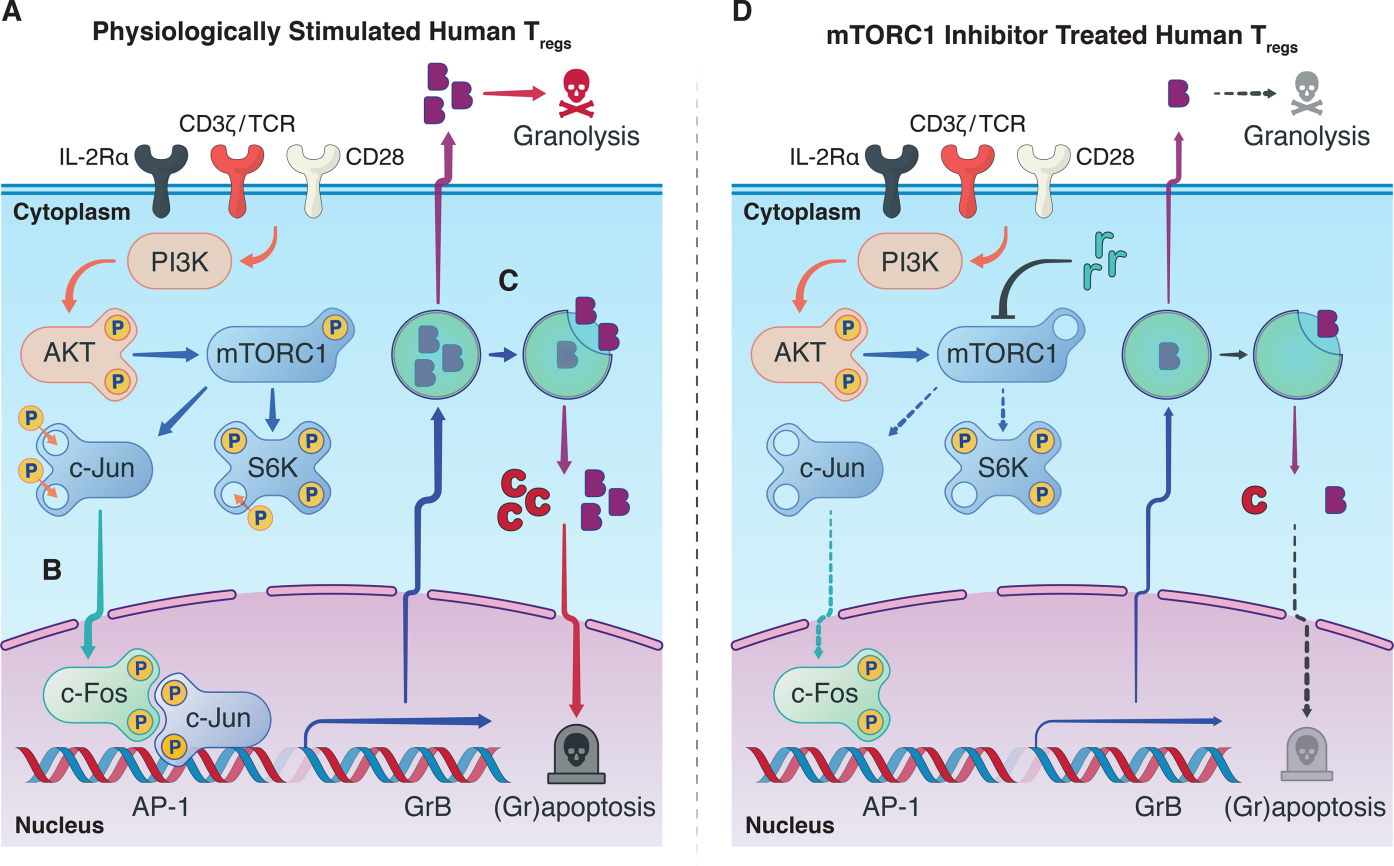
Observed interplay between mTORC1 and GrB pathways. **(A)** In a physiological setting, antigenic T_reg_ stimulation *via* the T-cell receptor, co-stimulatory CD28-based activation, and IL-2Rα (CD25)-dependent cytokine signaling, sets in motion a phosphorylation cascade involving phosphoinositide 3-kinase (PI3K), Akt, mTORC1, S6 kinase 1 (S6K), and c-Jun. Phosphorylated c-Jun is known to permit its nuclear translocation where it can undergo dimerization with p-c-Fos and generate transcription factor Activator Protein-1 (AP-1). **(B)** Our hypothesis is that AP-1 can unwind heterochromatic DNA in the promotor region of GrB in T_regs_ to boost the transcription of GrB mRNA. Conventionally, upon mRNA translation, the mature GrB proteins are stored in designated cytotoxic granules to save their potent cytolytic activity for target effector T cells. **(C)** In the context of protracted antigenic stimulation, however, GrB can inadvertently leak from its harboring lysosomes to precipitate caspase-dependent and independent cell death. **(D)** Treatment of T_regs_ with mTORC1 inhibitors such as rapamycin provide a multipronged effect, notably, decreasing phosphorylation of p-S6K and p-c-Jun, attenuating GrB expression and activity, and finally diminishing T_reg_ apoptosis. The exact biochemical constituents that drive this phenotypic and functional shift in the T_reg_ population upon mTORC1 inhibition remain to be elucidated and warrant further research efforts.

Synthesizing our findings with the knowledge garnered in the literature: Antigenic signatures are relayed to T_regs_ from the extracellular milieu through the T-cell receptor (CD3) with co-stimulatory (CD28) and cytokine (IL-2) signaling, activating host T_regs_ and mTORC1 (**Figure 4A**). We believe this permits a cascade of proliferative stimuli including S6K and c-Jun phosphorylation, resulting in the escalation of T_reg_ activation. Nuclear translocation of AP-1 heterodimers, including p-c-Jun, can then drive GrB transcription (**Figure 4B**), which can ultimately destabilize the autolysosome harboring synthesized stores of GrB and lead to their escape before being exocytosed (**Figure 4C**). In the same manner that GrB causes granolysis of target cells, nuclear translocation of GrB in host T_regs_ can cause granzyme-B-induced apoptosis—in part, explaining the poor homeostasis of adoptively transferred human T_regs_ through grapoptosis. Notably, we found that treatment of human T_regs_ with an mTORC1 inhibitor decreased S6K and c-Jun phosphorylation and attenuated both intracellular GrB expression and T_reg_ apoptosis (**Figure 4D**). Although further research efforts are warranted to deepen our understanding of the complex interplay between mTORC1 inhibition and grapoptosis, our findings provide preliminary insights towards another piece of the T_reg_ homeostasis puzzle.

### Limitations

As with all human T_reg_ studies, attaining large numbers of T_regs_ for *ex vivo* and *in vivo* experiments is restricted by methodological constraints, limiting the number of testable conditions. Development of alternative cell culture models and use of fluorescence-activated cell sorting methods would likely permit greater exploration of the exact mechanisms of action underpinning mTORC1-centric regulation of the human T_reg_ phenotype. Alternatively, murine models could be employed to decipher the specifics behind mTORC1 signaling and grapoptosis—even though human T_regs_ are a heterogeneous population of cells that are metabolically and phenotypically distinct cells from their murine counterparts (52–54).

Additionally, although the T_regs_ in this study were harvested from diverse donors, and each donor pool of human T cells responds differently to mitogenic stimuli (due to genetic and health factors pertaining to the donor), GrB was upregulated in all activated T_regs_, and this increase could consistently be neutralized with mTORC1 inhibition. Nonetheless, the clinical application of these findings, as with all human studies, remains to be validated in settings with clinically-relevant patient sample sizes.

## Conclusion

Extended activation of T_regs_ with antigenic, co-stimulatory, and cytokine signals upregulates a pro-apoptotic protein signature that includes the intracytoplasmic expression of granzyme B. Inhibition of mTORC1 in T_regs_ can neutralize this signature by in part decreasing the expression and activity of intracellular GrB, thus improving T_reg_ homeostasis. Whether mTORC1 inhibition solely decreases grapoptosis in host T_regs_ or also diminishes granolysis of target cells, warrants future research efforts.

## Data Availability Statement

The original contributions presented in the study are included in the article/**Supplementary Material**. Further inquiries can be directed to the corresponding authors.

## Ethics Statement

The studies involving human participants were reviewed and approved by Institutional Review Board at the Brigham and Women’s Hospital. Written informed consent for participation was not required for this study in accordance with the national legislation and the institutional requirements.

## Author Contributions

Authors are listed alphabetically: JRA formulated the overarching research aims. HA, JRA, and SE designed the experimental models and analyzed and interpreted the data. BD, GM, HA, IS, SE. performed experiments and collected data. BK, BP, JC provided study materials, reagents, patient samples, instrumentation, or other analysis tools. HA, JRA, and SE wrote the initial draft of the manuscript. AS, CD, JBA, JS, JL, LL, LVR, PC, and SB critically reviewed the manuscript and provided commentary. JRA, and SE revised the manuscript and prepared it for publication. JRA acquired funding for the work presented in the manuscript. All authors contributed to the article and approved the submitted version.

## Funding

This work was supported by the American Heart Association (AHA Award 13FTF17000018 to JRA), American Diabetes Association (ADA Award 1-17-IBS-206 to JRA), Qatar Foundation Grant (NPRP8-1744-3-357X to JRA), and the National Institutes of Health (RO1 AI134842 to JRA).

## Conflict of Interest

The authors declare that the research was conducted in the absence of any commercial or financial relationships that could be construed as a potential conflict of interest.

## Publisher’s Note

All claims expressed in this article are solely those of the authors and do not necessarily represent those of their affiliated organizations, or those of the publisher, the editors and the reviewers. Any product that may be evaluated in this article, or claim that may be made by its manufacturer, is not guaranteed or endorsed by the publisher.

## Supplementary Material

Supplementary Data Sheet 1 Original Data.
