# Supplementary material for: mTORC1 Inhibition Protects Human Regulatory T Cells From Granzyme-B-Induced Apoptosis
Source: Front Immunol. 2022 Jun 10;13:899975. doi: 10.3389/fimmu.2022.899975 (PMC9229986; doi:10.3389/fimmu.2022.899975)
Supplement: Supplementary file 2 [file DataSheet_2.docx]

Supplementary Materials

# Supplementary Methods

## Mice

*NOD.Cg-Prkdc^scid^ Il2rg^tm1Wjl^/Sz* (NSG, #005557) mice were obtained from The Jackson Laboratories (Bar Harbour, ME, United States) and were maintained in specific-pathogen free conditions. Mice were age- and sex-matched. The study protocol was approved by the Brigham and Women’s Hospital Institutional Animal Care and Use Committee (IACUC).

## Isolation of cells

PBMCs were isolated within 24 hours using SepMate tubes (#85450, STEMCELL Technologies) and Lymphoprep density gradient medium centrifugation at 800× *g* for 30 minutes. Human T_regs_ and conventional T cells (CD4^+^CD25^hi^CD127^lo^ T_regs_ and CD4^+^CD25^-^ T_cons_, respectively) were isolated from leukapheresed peripheral blood of a healthy donor using either a MACS-based (#130-094-775, Miltenyi Biotec) or EasySep-based (#18063, Stemcell Technologies) approach as per the manufacturer’s instructions.

## Humanized skin transplant model

Donated human skin allografts from healthy donors undergoing cosmetic surgery were harvested and processed using blunt-tipped forceps. Fur of recipient NOD-*scid* IL-2 receptor-γ^null^ (NSG) mice were shaved, 1.0-1.5 cm^2^ of skin was excised from the dorsal trunk, and the processed donor skin was sutured with PERMA-HAND 4-0 Silk Suture (#1677G, Ethicon), allowing it to engraft for seven days. Skin grafts were secured in place with dry gauze and bandaged.

## Adoptive T_reg_ transfer model

For the humanized adoptive transfer model, 5.0×10^6^ peripheral blood mononuclear cells (PBMCs) and 1.0×10^6^ T_regs_ were isolated within 24 hours of healthy human donations, as described above, and injected retro-orbitally in 90-μL total volumes of 1× Dulbecco’s phosphate-buffered saline (DPBS) at day seven post-transplant. Starting on the day of the adoptive transfer, the mice were intraperitoneally injected once daily for nine days with 200-μL volumes of either 1× DBPS or 1 mg/kg rapamycin. The mice were necropsied for mechanistic studies at day fourteen post adoptive transfer, which was twenty-one days following the skin graft transplantation.

## Quantification of granzyme B activity

Granzyme B activity within target cells was measured using the GranToxiLux assay in which a membrane permeable GrB-sensitive substrate is cleaved by intracytoplasmic GrB and can be detected using surrogate green fluorescence emission. The assay was purchased from OncoImmunin (Gaithersburg, MD, USA) and performed as per the manufacturer’s instructions.

## Flow cytometry

Cells for flow cytometric analysis were stained extra- and intracellularly using the eBioscience FoxP3/Transcription Factor Staining Buffer Set (#00-5523-00, Invitrogen), and the stained cells were analyzed on a FACS Canto II (BD Biosciences, San Jose, CA, United States) flow cytometer. The resultant files were analyzed with FlowJo (Version 10; Flowjo LLC, Ashland, OR, United States).

Isolated human cells were stained with: Anti-4E-BP-FITC (P-1, 1:125 μL; # sc-9977, Santa Cruz Biotechnology), Annexin V-BV510 (1:125 μL; #640937, BioLegend), Annexin V-PE (1:125 μL; #640907, BioLegend), anti-CD127-PE/Cy7 (A019D5, 1:250 μL; #351319, BioLegend), anti-CD25-APC/Cy7 (M-A251, 1:500 μL; #356121, BioLegend), anti-CD25-PE (M-A251, 1:500 μL; #356103, BioLegend), anti-CD4-APC/Cy7 (OKT4, 1:500 μL; #317417, BioLegend), anti-CD4-BV510 (RPA-T4, 1:500 μL; #300545, BioLegend), anti-CD4-FITC (OKT4, 1:500 μL; #317407, BioLegend), anti-FoxP3-APC (PCH101, 1:125 μL; #17477642, Invitrogen), anti-Granzyme-B-Pacific Blue (GBII, 1:62.5 μL; #515407, BioLegend), anti-p-c-Jun-FITC (KM-1, 1:125 μL; #sc-822, Santa Cruz Biotechnology), and anti-p-p70-S6-Kinase-α-AF647 (A-6, 1:125 μL; #sc-8416, Santa Cruz Biotechnology).
