# Supplementary material for: mTORC1 Inhibition Protects Human Regulatory T Cells From Granzyme-B-Induced Apoptosis
Source: Front Immunol. 2022 Jun 10;13:899975. doi: 10.3389/fimmu.2022.899975 (PMC9229986; doi:10.3389/fimmu.2022.899975)
Supplement: Supplementary file 3 [file DataSheet_3.docx]

Supplementary Materials

# Supplementary Figures

**
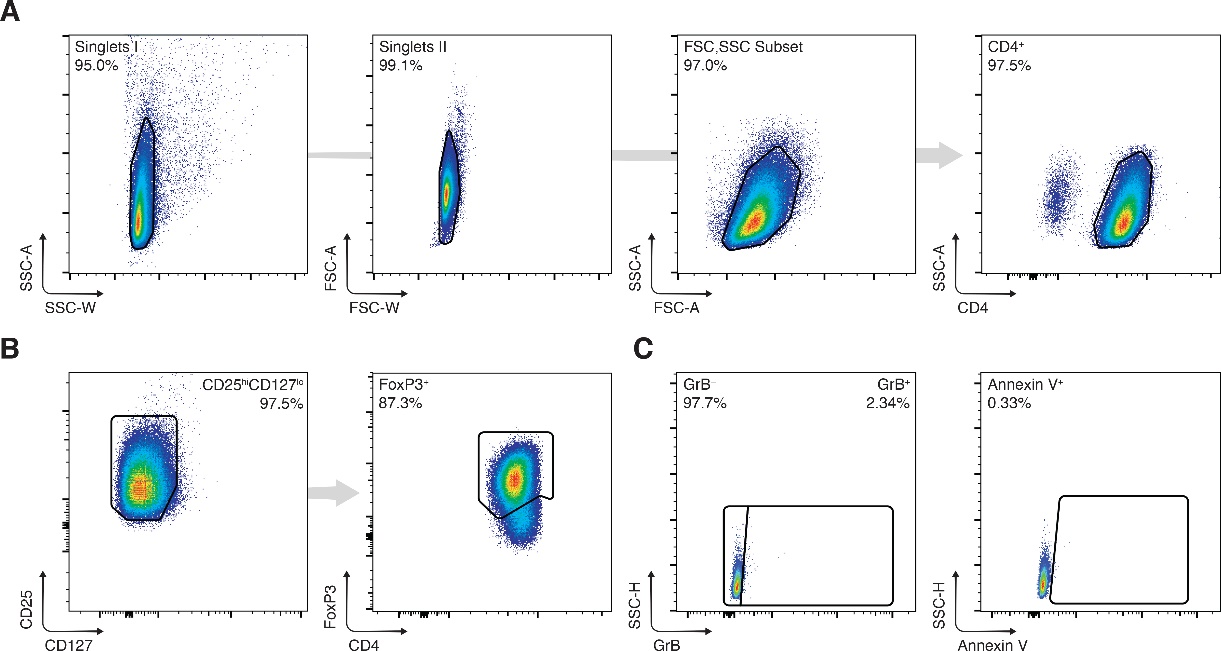
**

**Figure S1** *Magnetic-activated CD4^+^CD25^+^CD127^-^ cell sorting purity of human T_regs_.* (**A** to **C**) Isolation purity of magnetically sorted CD4^+^ (A) CD25^hi^CD127^lo^ FoxP3^+^ (B) human T_regs_ with granzyme B and annexin V expression levels (C) immediately post isolation. GrB, granzyme B.


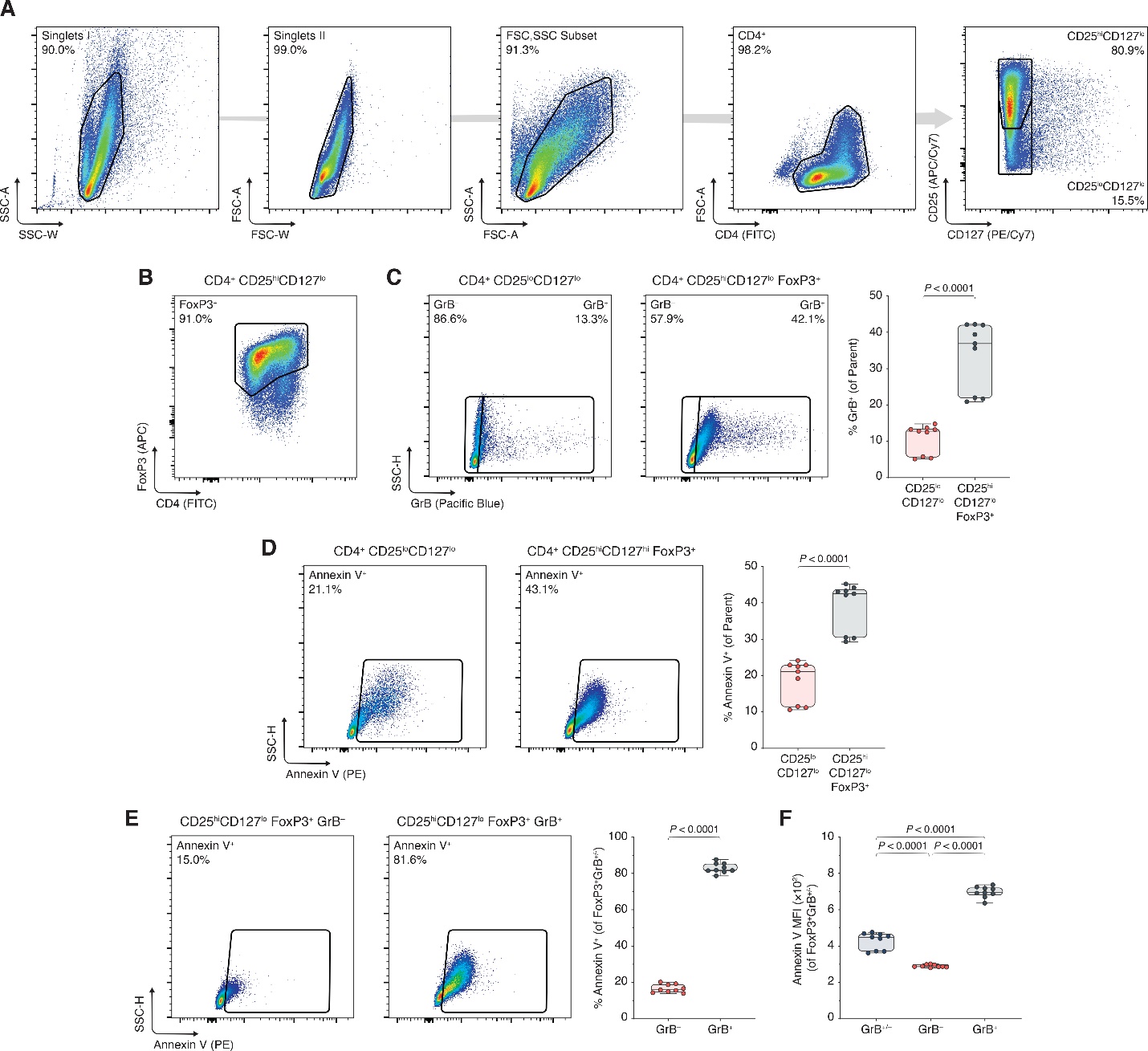


**Figure S2** *Human T_reg_ phenotyping strategy and activation-induced upregulation of GrB and apoptosis.* Human T_regs_ were isolated and expanded for three days with α-CD3, α-CD28, and IL-2 (n = 3 technical replicates/condition; 3 representative experiments). (**A** to **B**) Gating strategy identifying magnetically sorted, activated human CD4^+^ CD25^hi^CD127^lo^ T cells (A), and therein the FoxP3^+^ subset (B). (**C**) Flow cytometric analysis of GrB^+^ cells among the CD4^+^ CD25^lo^CD127^lo^ T cell and CD4^+^ CD25^hi^CD127^lo^ FoxP3^+^ T_reg_ subsets and accompanying box plots. (**D**) Flow cytometric analysis and box plots of Annexin V^+^ cells among the CD4^+^ CD25^lo^CD127^lo^ T cell and CD4^+^ CD25^hi^CD127^lo^ FoxP3^+^ T_reg_ subsets. (**E**) Flow cytometric analysis of Annexin V^+^ cells among GrB^-^ and GrB^+^ FoxP3^+^ T_reg_ subsets, including box plots. (**F**) Box plots of Annexin V MFI among total GrB (GrB^+/-^), GrB^-^, and GrB^+^ FoxP3^+^ T_reg_ subsets. Data represent boxplots with median, interquartile range, minimum, maximum, and all individual data points of the denoted experimental groups. *P* values were calculated with independent samples two-tailed Student’s *t*-tests, and non-parametric Mann-Whitney *U*-tests were performed when the assumption of homoscedasticity could not be met. For analyses with more than two groups, one-way analyses of variance followed by Holm-Šídák multiple comparison tests were performed. GrB, granzyme B; MFI, mean fluorescence intensity.


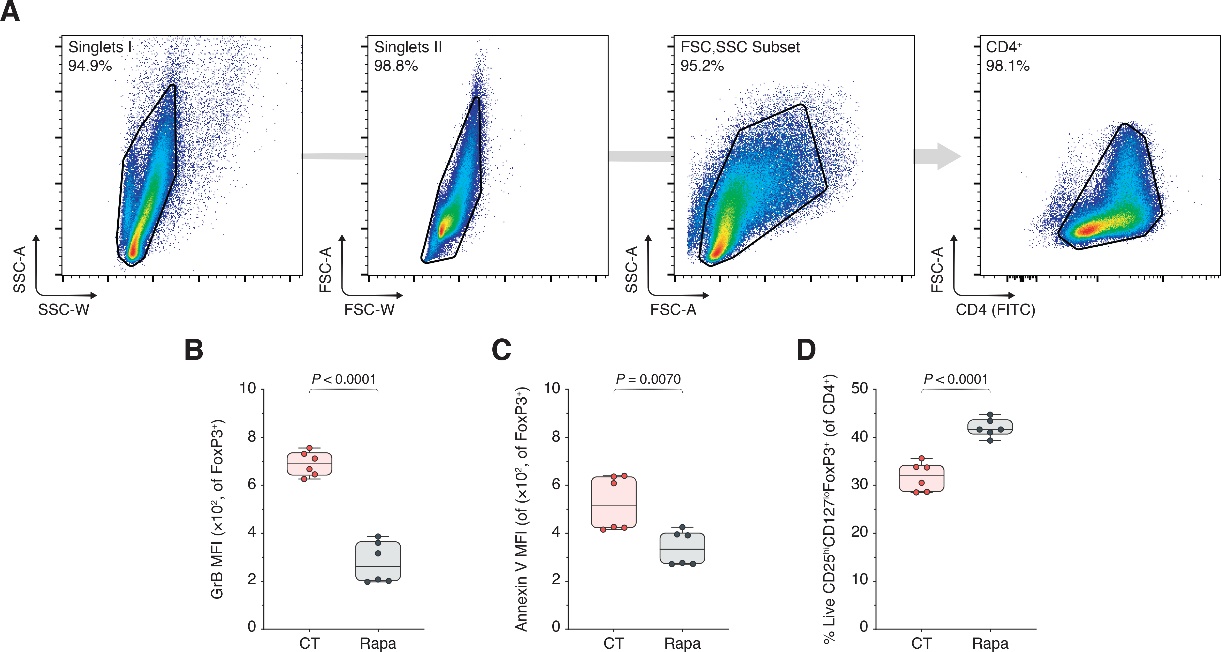


**Figure S3** *Rapamycin experiments phenotyping strategy and supplementary box plots related to figure 1.* (**A**) Gating strategy identifying magnetically sorted, activated human CD4^+^ T cells. (**B** to **D**) Box plots of GrB (B) and Annexin V (C) mean fluorescence intensities (MFIs), and percentages of live Annexin V^-^ CD25^hi^CD127^lo^ FoxP3^+^ T_regs_ (D) among CD4^+^ T cells (n = 3 technical replicates/condition; 2 representative experiments of 5). Data represent boxplots with median, interquartile range, minimum, maximum, and all individual data points of the denoted experimental groups. *P* values were calculated with independent samples two-tailed Student’s *t*-tests, and non-parametric Mann-Whitney *U*-tests were performed when the assumption of homoscedasticity could not be met. CT, control; GrB, granzyme B; MFI, mean fluorescence intensity; Rapa, rapamycin.


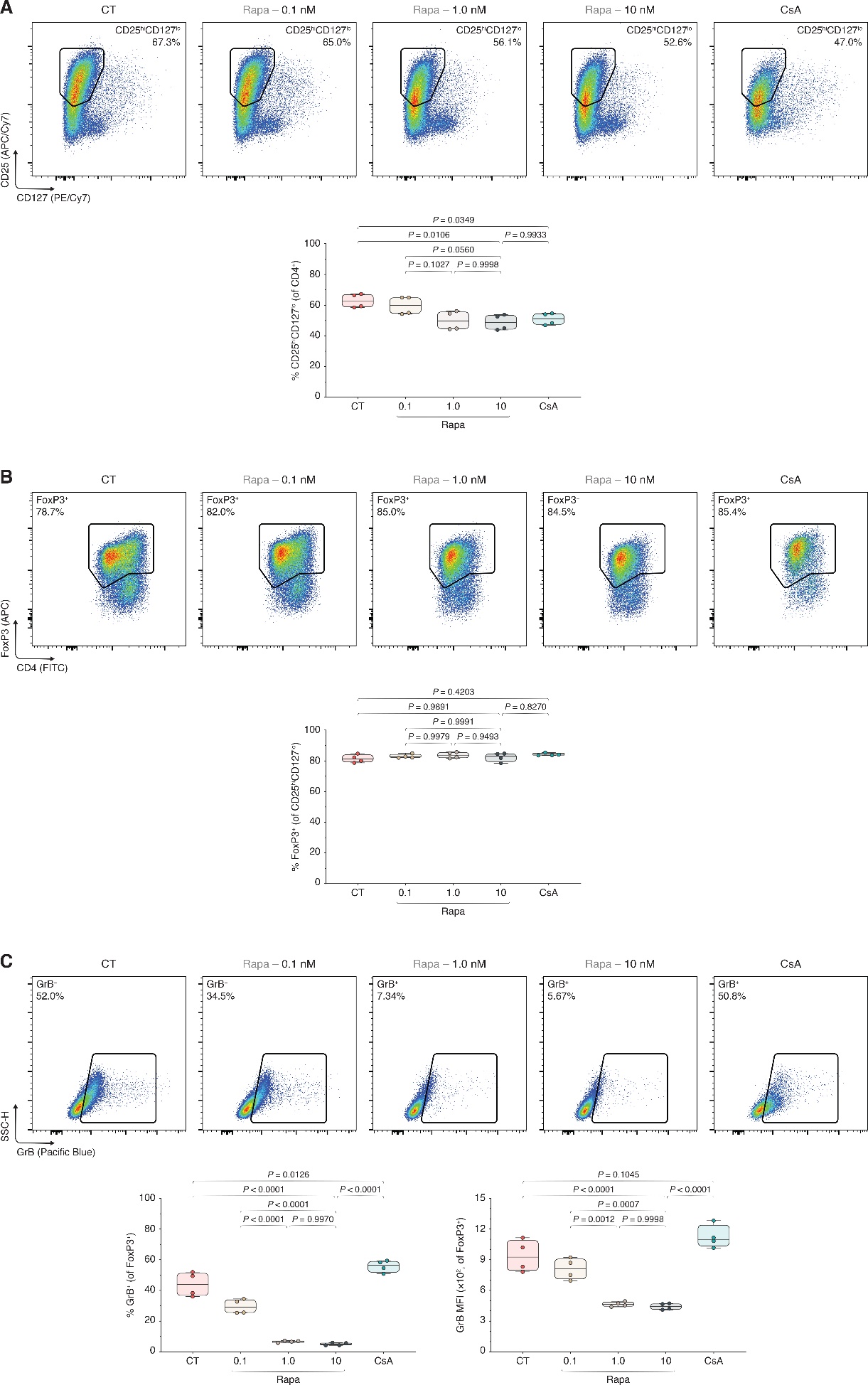


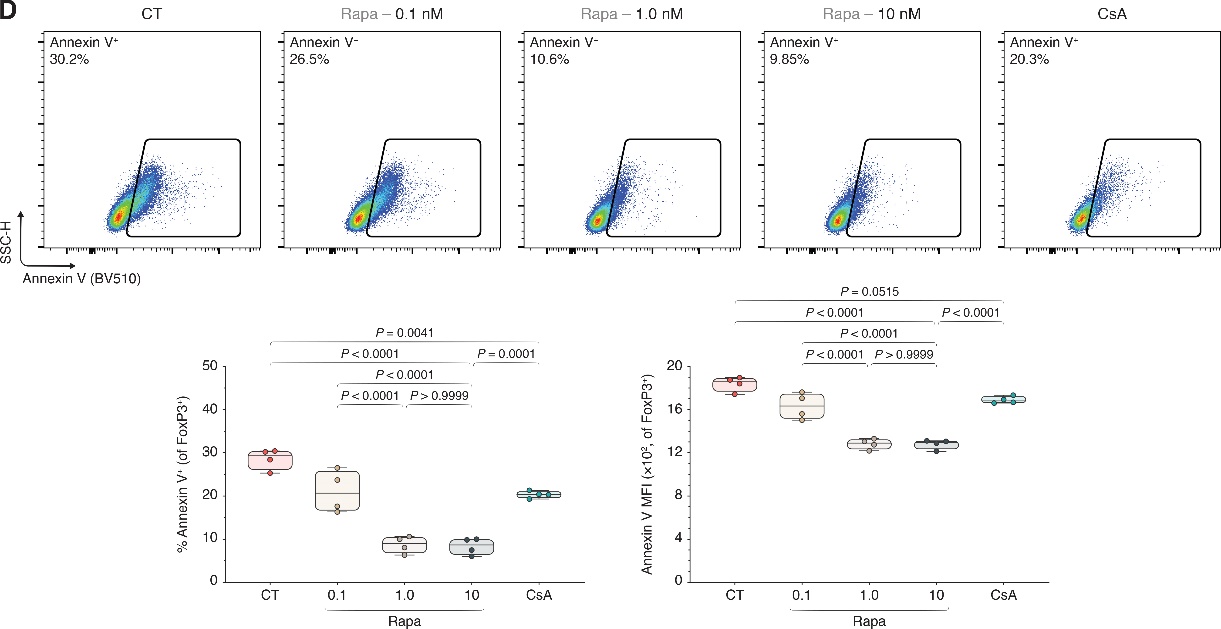


**Figure S4** *Rapamycin dose de-escalation increases GrB expression and apoptosis in human T_regs_ and affects T_reg_ viability discordantly from calcineurin inhibition.* Human T_regs_ were isolated and expanded for three days with α-CD3, α-CD28, and IL-2, without additional inclusions (CT), with CT stimulants plus 0.1–10 nM rapamycin (Rapa), or with CT stimulants and cyclosporin A (CsA). (**A** to **D**) Flow cytometric analyses of CD25^hi^CD127^lo^ subset among CD4^+^ T cells (A), FoxP3^+^ subset among CD25^hi^CD127^lo^ cells (B), and GrB^+^ (C) and Annexin V^+^ subsets (D) among FoxP3^+^ T_regs_, including the respective box plots (n = 2 technical replicates/condition; 2 experiments). Data represent boxplots with median, interquartile range, minimum, maximum, and all individual data points of the denoted experimental groups. *P* values were calculated with one-way analyses of variance followed by Holm-Šídák multiple comparison tests. CsA, cyclosporin A; CT, control; GrB, granzyme B; MFI, mean fluorescence intensity; Rapa, rapamycin.


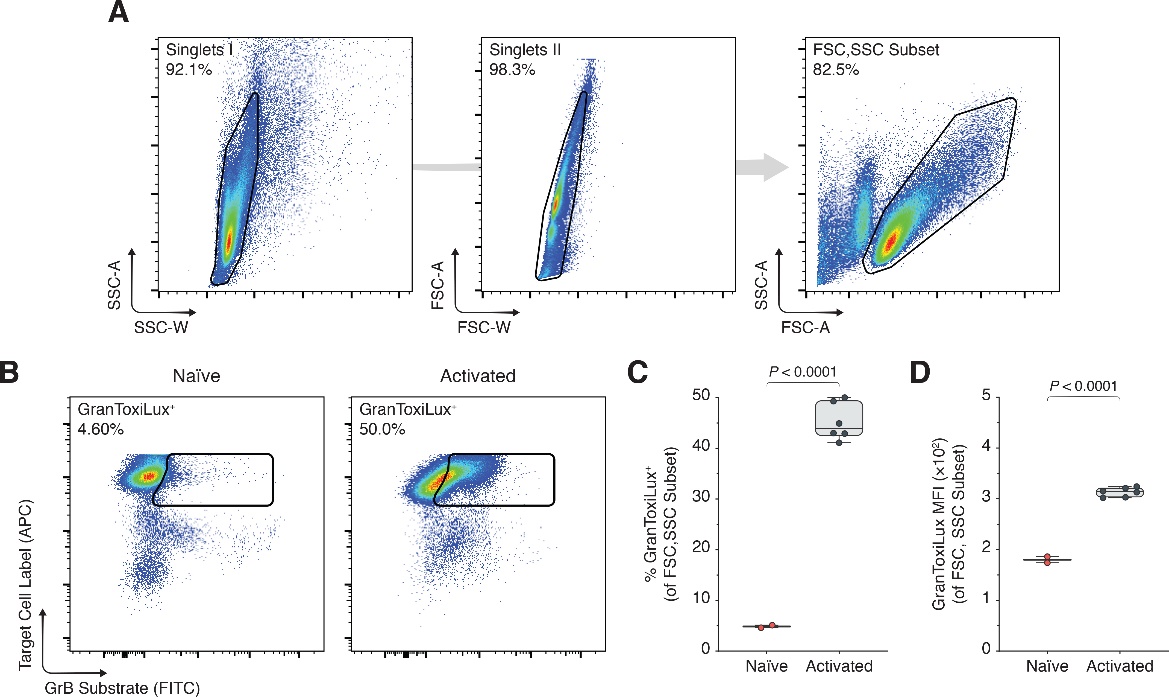


**Figure S5** *GranToxiLux experiments phenotyping strategy and T_reg_ stimulation leads to intracytoplasmically active GrB.* (**A**) Gating strategy identifying an active FSC^hi^SSC^hi^ subset of magnetically sorted CD4^+^ CD25^hi^CD127^lo^ human T_regs_ that were expanded for three days with α-CD3, α-CD28, and IL-2. (**B** to **D**) Flow cytometric analysis and box plots of GranToxiLux^+^ T_reg_ percentages (B and C), and GranToxiLux mean fluorescence intensities (E; n = 3 technical replicates/condition; 2 representative experiments of 4). Data represent boxplots with median, interquartile range, minimum, maximum, and all individual data points of the denoted experimental groups. *P* values were calculated with independent samples two-tailed Student’s *t*-tests, and non-parametric Mann-Whitney *U*-tests were performed when the assumption of homoscedasticity could not be met. GrB, granzyme B; MFI, mean fluorescence intensity.


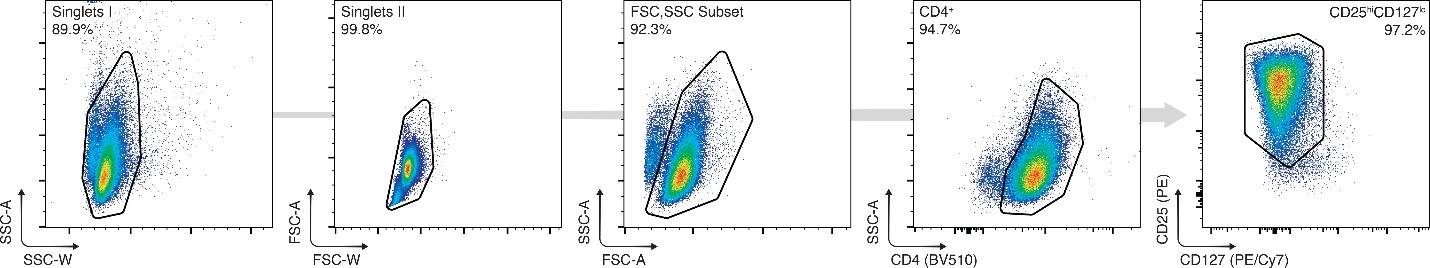


**Figure S6** *Phosphorylation experiments* *phenotyping strategy.* Gating strategy identifying magnetically sorted CD4^+^ CD25^hi^CD127^lo^ human T_regs_ that were expanded in a 24-hour cell culture window with α-CD3, α-CD28, and IL-2.


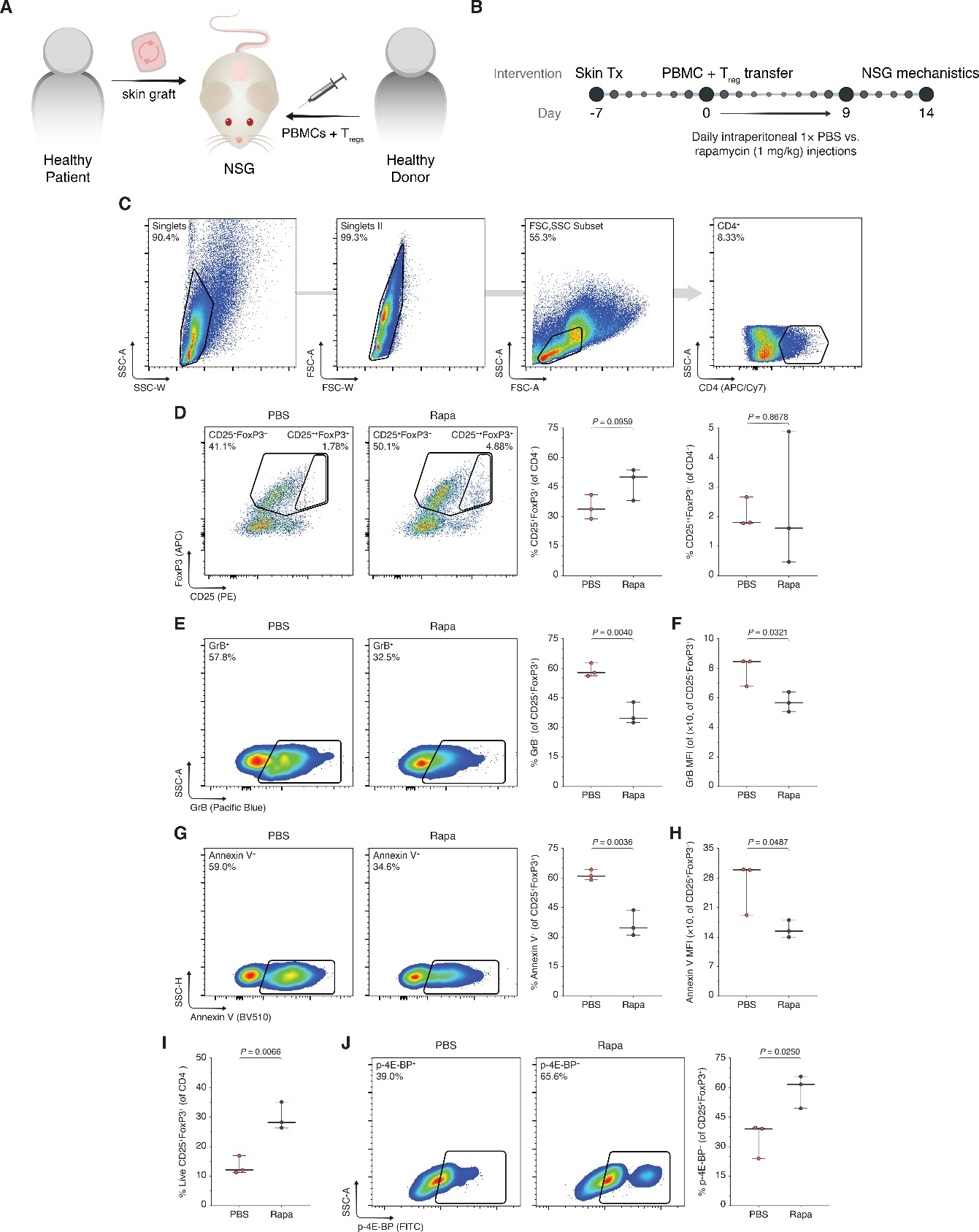


**Figure S7** *In vivo mTORC1 inhibition reduces human T_reg_ GrB expression apoptosis of peripheral T_regs_ in an in vivo allotransplantation model.* (**A** and **B**) NOD-*scid* IL-2 receptor-γ^null^ (NSG) mice were transplanted with healthy donor skin seven days prior to the adoptive transfer of 5.0×10^6^ human PBMCs and 1.0×10^6^ human CD4^+^CD25^hi^CD127^lo^ T_regs_ (A). Afterwards, the NSG mice received daily intraperitoneal injections of 1× DPBS (PBS) or 1 mg/kg rapamycin (Rapa) for nine days. Fourteen days from the start of the treatment, the mice were euthanized and the splenocytes were extracted (B; n = 3 mice/condition; 1 of 2 experiments). (**C**) Gating strategy identifying splenic CD4^+^ human T cells. (**D**) Flow cytometric analysis and box plots of CD25^+^FoxP3^+^ and CD25^++^FoxP3^+^ T_regs_ among CD4^+^ T cells. (**E** and **F**) Flow cytometric analysis and box plots of GrB^+^ cells among FoxP3^+^ T_regs_ (E), and box plots of GrB mean fluorescence intensities (MFIs) among FoxP3^+^ T_regs_ (F). (**G** and **H**) Flow cytometric analysis and box plots of Annexin V^+^ cells among FoxP3^+^ T_regs_ (G), and box plots of Annexin V MFIs among FoxP3^+^ T_regs_ (H). (**I**) Box plots of live Annexin V^-^ CD25^hi^CD127^lo^ FoxP3^+^ T_regs_ among CD4^+^ T cells. (**J**) Flow cytometric analysis and box plots of p-4E-BP^+^ cells among FoxP3^+^ T_regs_. Data represent boxplots with median, interquartile range, minimum, maximum, and all individual data points of the denoted experimental groups. *P* values were calculated with independent samples two-tailed Student’s *t*-tests, and non-parametric Mann-Whitney *U*-tests were performed when the assumption of homoscedasticity could not be met. GrB, granzyme B; MFI, mean fluorescence intensity; Rapa, rapamycin.
